# Tribological Properties of Eutectic White Cast Iron with Directional and Non-Directional Microstructure

**DOI:** 10.3390/ma18194516

**Published:** 2025-09-28

**Authors:** Małgorzata Trepczyńska-Łent, Jakub Wieczorek

**Affiliations:** 1 Faculty of Mechanical Engineering, Bydgoszcz University of Science and Technology, Al. Prof. S. Kaliskiego 7, 85-796 Bydgoszcz, Poland; 2 Faculty of Materials Engineering, Silesian University of Technology, Krasińskiego 8 St., 40-019 Katowice, Poland; jakub.wieczorek@polsl.pl

**Keywords:** eutectic white cast iron, directional solidified structure, tribological properties, coefficient of friction, wear

## Abstract

Tribological tests were conducted on eutectic white cast iron subjected to directional solidification (resulting in a directionally oriented microstructure) and, for comparison, on white cast iron with an equiaxed (non-directional) structure. The tests were performed under dry sliding conditions on a pin-on-block rig using Cu, AlSi12CuNiMg alloy, AlSi12CuNiMg + SiC composite, and steel grade 1.3505. The friction coefficient and wear rates of these materials were systematically compared. Quantitative and qualitative evaluations of the wear tracks formed on the test specimens were carried out using profilometry. The results demonstrate that the directionally solidified white cast iron exhibits improved friction coefficient stability and reduced wear in the specific tribological pairs. The oriented directional structure demonstrated more favourable interactions when paired with AlSi12CuNiMg + SiC composite and 1.3505 steel. These tribological combinations exhibited reduced roughness values across selected cross-sectional analyses, resulting in correspondingly lower Sa parameter measurements. This finding suggests a promising new application for inserts made of directionally structured white cast iron in structural components requiring enhanced wear resistance at elevated temperatures.

## 1. Introduction

Material strength and the complex of functional properties resulting from manufacturing technology and related to material surface characteristics are the decisive factors determining the operational lifespan of machines and equipment. The issues concerning surface condition changes and modifications of the surface layer in machine components are difficult to generalize. This complexity stems from the intricate nature of processes occurring at the material surface and their dependence on numerous variables [1,2,3]. Even more challenging is the unambiguous definition of friction and wear phenomena in relation to materials with multiphase structure. Among critical considerations is the determination of which microstructural features most significantly influence the tribological properties of a given material.

Foundry alloys have found extensive application in machine components that operate under friction conditions. There exists a continuous need for searching and investigating suitable materials for friction pairs. Multiphase materials such as cast iron, including white cast iron, are also considered in this context. Over several decades, white cast irons have served as the primary materials for wear protection applications in mining and cement industries, as well as in road construction. Advanced wear-resistant metal matrix composites have been developed, incorporating hard particles such as carbides, borides, and nitrides within a metal matrix, designed with microstructures exhibiting superior properties. The selection of cast iron for friction pairs must consider its chemical composition, microstructure, and casting technology. The abrasive wear resistance of white cast iron depends primarily on the properties and proportion of individual phases and their mutual distribution. Extensive research has been conducted on the abrasive wear resistance of alloyed white cast iron [4,5,6,7,8,9,10,11,12,13,14,15,16,17,18,19,20].

The microstructure of high-chromium white cast iron often features a columnar zone where eutectic carbides align with the direction of heat dissipation, resulting in a distinctly anisotropic morphology. Research employing a pin abrasion test on specimens from this zone investigated the wear behaviour in directions both parallel (longitudinal) and perpendicular (transverse) to the primary axis of the carbides. The findings indicated that longitudinally oriented specimens exhibited a 27–67% lower volume loss compared to their transverse counterparts, with the exact reduction depending on the specific heat treatment applied [21]. The M_7_C_3_ carbide is a chromium-rich compound where M denotes various metals such as chromium, iron, vanadium, molybdenum, and tungsten. The relative proportions of these metallic elements within the carbide can vary depending on the specific alloy composition and the processing parameters applied [22]. The influence of M_7_C_3_ carbide orientation in high-chromium white cast irons was explored using high-stress pin-on-drum abrasion and single-scratch tests. This investigation concluded that orienting the long axes of the carbides perpendicular to the wear surface significantly compromises abrasion resistance. Conversely, superior wear resistance was achieved when the carbides were aligned parallel to the wear surface, with optimal performance observed when this parallel alignment was also oriented perpendicular to the direction of sliding [23]. In another study, two white cast iron alloys with differing chromium contents (13.81% and 24.41%) were unidirectionally solidified to produce oriented M_7_C_3_ carbides within a eutectic matrix. Pin-abrasion tests on specimens sectioned parallel and transverse to the solidification axis yielded contrasting results, revealing that the transverse sections demonstrated greater abrasion resistance. The authors attributed this behaviour to the fracture mechanics of M_7_C_3_ carbides, where cracks tend to propagate along their longest edge, which corresponds to the plane of lowest fracture toughness [24].

Unalloyed, eutectic white cast iron is characterized by the presence of transformed ledeburite in its microstructure. Such cast iron can be treated as an in situ composite. This composite consists of a eutectic mixture of cementite (iron carbide Fe_3_C) in the form of layers (plates) and pearlite in the form of fibres (rods). Cementite hardness ranges from approximately 760–1340 HV [25], while pearlite hardness averages 180–220 HB. Hard cementite in this composite is responsible for load transfer, while pearlite provides plastic properties. Ledeburite hardness is approximately 450 HB.

Research has demonstrated that in carbon steel and white cast iron, the volumetric fraction of cementite significantly influences abrasive wear resistance. Nevertheless, since the cementite volume fraction in conventional steels and cast irons remains below 65%, the wear behaviour of Fe-C materials containing cementite volume fractions exceeding 65% remains largely unexplored [25]. Investigations have been conducted on abrasive wear behaviour using materials with varying cementite volume fractions [26].

Due to microstructural characteristics and properties, white cast iron is used to manufacture components resistant to abrasive wear, as well as artifacts requiring high resistance to high and low temperature effects (camshafts, extrusion dies, pipe couplings, flanges, pump impellers, shot blast nozzles). The production of inserts (fragments) or surfaces and coatings made of white cast iron on structural elements of other metallic materials is also popular. Industrial applications frequently employ cladding techniques for specific surface areas exposed to severe wear conditions as a cost-effective solution for extending component service life. Among various surface coating protection and hardening techniques, hardfacing represents one of the most widely adopted approaches due to its economic advantages and operational simplicity [19,26,27,28,29].

The installation method of a given white cast iron insert may be significant depending on the operational mode of the structural element. In such cases, the possibility of using white cast iron with directional microstructure appears important. The mechanical properties of such cast iron depend on the working direction relative to directional solidification. The authors’ previous studies [30,31,32,33] examined the microstructure, microhardness, and strength of directionally solidified eutectic white cast iron. Directional solidification involves the controlled removal of heat from the interface between the liquid and solid phases, as well as the controlled advancement of the melting or solidification front along the sample. This process enables the production of materials with precisely tailored properties. Building upon the author’s research, investigating the abrasive wear resistance of eutectic white cast iron presents an interesting avenue. A hypothesis can be formulated regarding the beneficial influence of microstructure obtained through directional solidification of white cast iron on its tribological properties. It is important to compare, for example, the abrasion resistance of white cast iron having the same chemical composition, but differing microstructures obtained through different casting technologies. A directional structure can be achieved through directional solidification, for instance, using a Bridgman apparatus, whereas a non-directional structure results from casting methods where volumetric solidification occurs.

## 2. Material and Research Methodology

### 2.1. Directional Solidification of White Cast Iron

**Figure 1:**
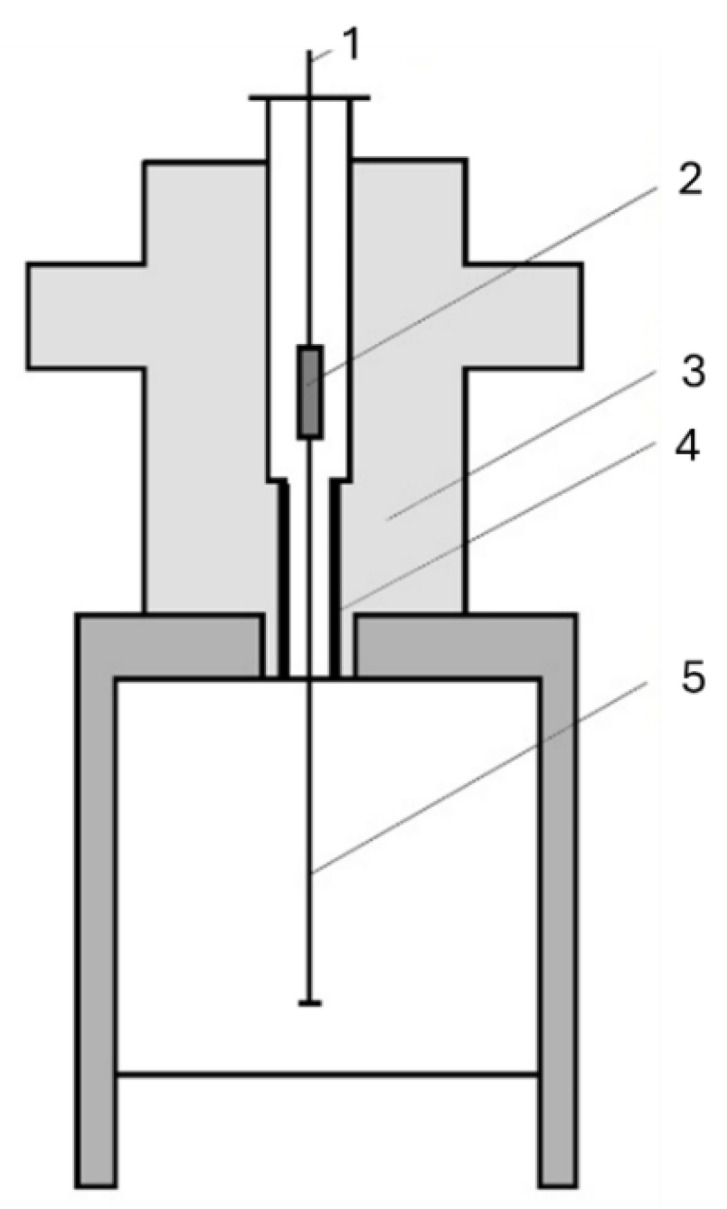
Schematic illustration of experimental setup in the Bridgman apparatus: 1-thermocouple, 2—sample, 3—heating furnace, 4—cooling system, 5—pulling system [30].

Directional solidification of cast iron was performed using a Bridgman apparatus with vertical temperature gradient (Faculty of Foundry Engineering, AGH, Krakow, Poland). Initially, Fe-C alloy ingots with a diameter of 12 mm were prepared in an argon atmosphere using a Balzers-type furnace, employing Armco iron and pressed graphite of spectral purity 99.99% C. Subsequently, cylindrical bars of the Fe-C alloy with a diameter of 5 mm were directionally solidified in a Bridgman apparatus using an alundum crucible under argon atmosphere. After maintaining the melt at 1450 °C for a homogenization period, the molten alloy was withdrawn downward from the heating zone directly into the cooling section at a constant withdrawal rate of 167 μm/s (equivalent to 600 mm/h), maintaining a steady temperature gradient of 33.5 K/mm [30,31,32,33]. Figure 1 shows a schematic illustration of experimental setup in the Bridgman apparatus.

**Figure 2:**
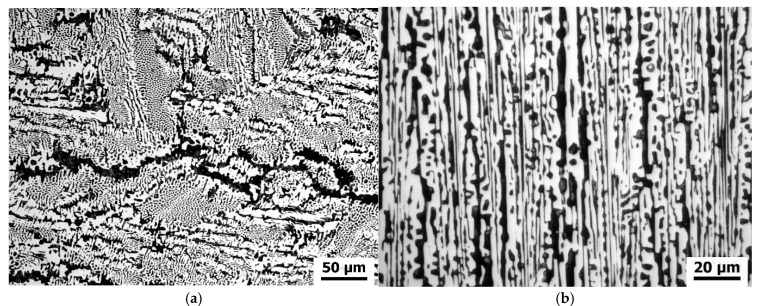
Microscopic images of: (**a**) non-directional structure; (**b**) directional structure white eutectic cast iron.

The chemical composition of the eutectic cast iron used is presented in Table 1. The chemical composition was determined using a SPECTROMAXX spectrometer (Spectro Analytical Instruments, Kleve, Germany). Figure 2 shows the non-directional and directional microstructure of white cast iron, whose structural component is transformed ledeburite. In Figure 2a, a pronounced equiaxed dendritic growth of the ledeburite is visible. In contrast, Figure 2b shows alternating cementite plates and pearlite regions (within the transformed ledeburite), which are parallel to each other and to the axis of the sample (the pulled rod).

### 2.2. Tribological Investigations

Tribological investigations were conducted at the Faculty of Materials Engineering, Silesian University of Technology in Katowice. Testing of tribological properties, including determination of friction coefficient values and abrasive wear resistance, was performed at room temperature (25 ± 1 °C) under technically dry friction conditions using a specimen (block)—counter-sample (pin) configuration. A T-01 block-on-pin tribological tester operating with reciprocating motion at 12 mm stroke was utilized for tribological property determination. The counter-specimen in the form of a pin with 5 mm diameter and 20 mm length was manufactured from eutectic white cast iron. Two pins were employed: one with directional structure (resulting from directional crystallization) and one with non-directional structure. Results were compared based on the applied white cast iron pin structure.

Four materials with varied mechanical properties (such as hardness, ductility, and tensile strength) were employed as blocks in friction pairs for tribological testing, paired with cast iron pins featuring non-directional and directional structures. The investigations were conducted using the following block materials (counter-specimens): electrolytic copper (ISO Cu-ETP [34], PN M1E), aluminum alloy AlSi12CuNiMg (EN AC-48000, [35]), composite based on AlSi12CuNiMg aluminum alloy (EN AC-48000 [35]) reinforced with SiC (reinforcing particle volume fraction 15%, diameter 15 µm), and bearing steel 1.3505 (100Cr6, [36]). The chemical composition of these materials is presented in Table 2. Composition of these alloys was tested by mass spectrometry (Foundry Master, Oxford Instruments—WAS, Abingdon, UK).

**Figure 3:**
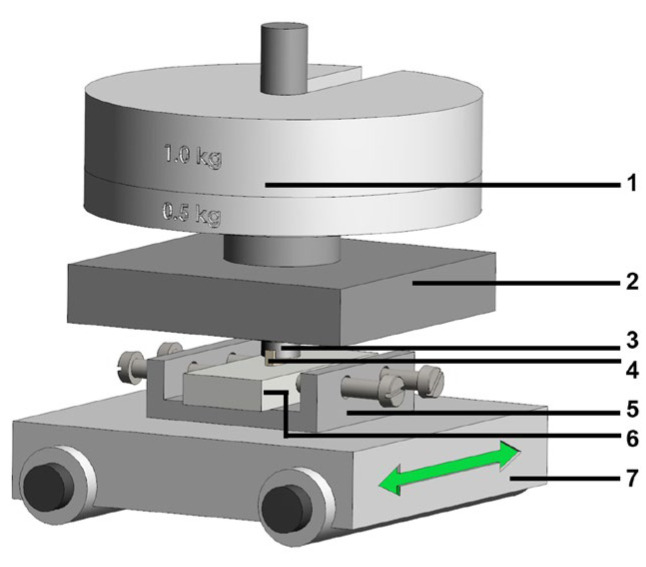
Schematic representation of the experimental setup used for tribological testing in pin-on-block configuration. The components are labelled as follows: 1—applied load, 2—strain gauge holder, 3—sample holder for the pin, 4—cast iron pin, 5—sample holder for the block, 6—sample, and 7—movable plate, green arrows—direction of sample movement [37].

Block surfaces were ground using corundum abrasive papers to 500 grit. The prepared surfaces were subjected to abrasion. The experimental pin-on-block setup used in tribological tests is shown in Figure 3. A load of 0.5 MPa unit pressure was applied through the pins, the sliding speed was 0.5 m/s, and the friction distance was 200 m. During testing, continuous recording of friction coefficient value changes was performed. The wear trace formed on the block surfaces was subjected to profilometric analysis. Surface structure analysis after friction of blocks and cast iron pins was conducted based on profilometric measurements performed using an FRT MicroProf profilometer with WCL3000 aberration head (FRT GmbH, München, Germany). This measurement is contactless, completely eliminating the possibility of wear trace deformation during testing. Wear trace geometry investigations were conducted immediately after the friction process completion. The only surface preparation before measurement was cleaning with ethanol solution. Parameters of the conducted surface geometry measurements were:

- measurement field 6 × 10 mm,
- resolution 600 × 1000 measurement points,
- data acquisition and processing—MARK III v3.8.21 for windows software (FRT GmbH, München, Germany).

The analysis employed two- and three-dimensional images to evaluate basic surface characteristics such as: wear trace depth, roughness and waviness in the friction area, and wear trace volume loss. Macroscopic photographs of wear traces were taken using a Nikon 7000 digital camera (Tokyo, Japan).

## 3. Results and Analysis

### 3.1. Profilometry of Wear Tracks

**Figure 4:**
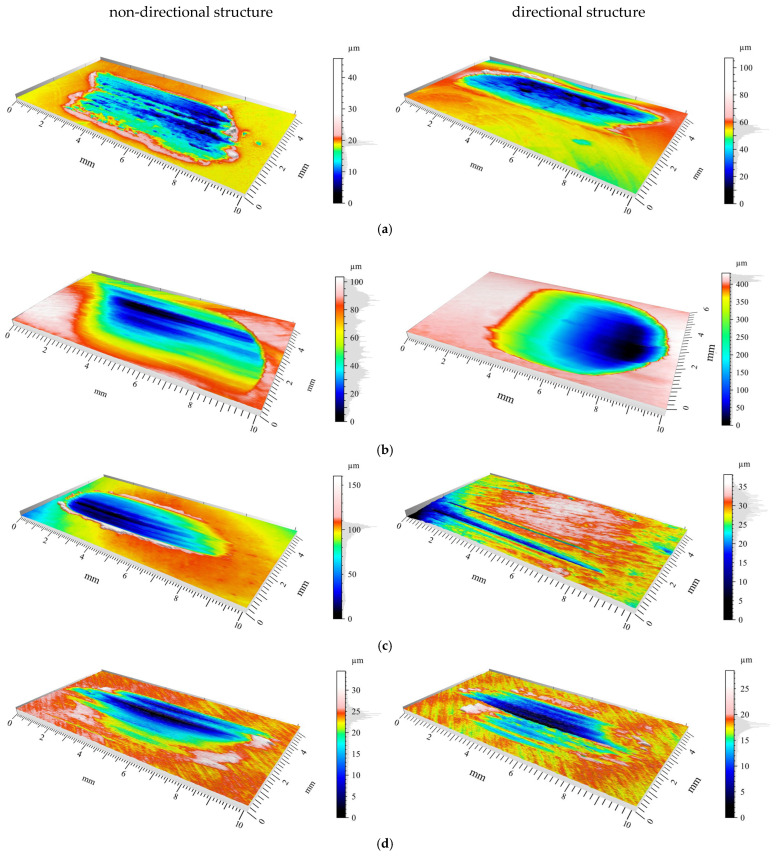
Three-dimensional surface topography images of wear tracks: (**a**) Cu-ETP; (**b**) AlSi12CuNiMg alloy; (**c**) AlSi12CuNiMg + SiC composite; (**d**) 1.3505 steel.

Figure 4 presents images of sliding traces after tribological tests on blocks of four materials obtained after cooperation with cast iron pins having non-directional and directional structures.

**Figure 5:**
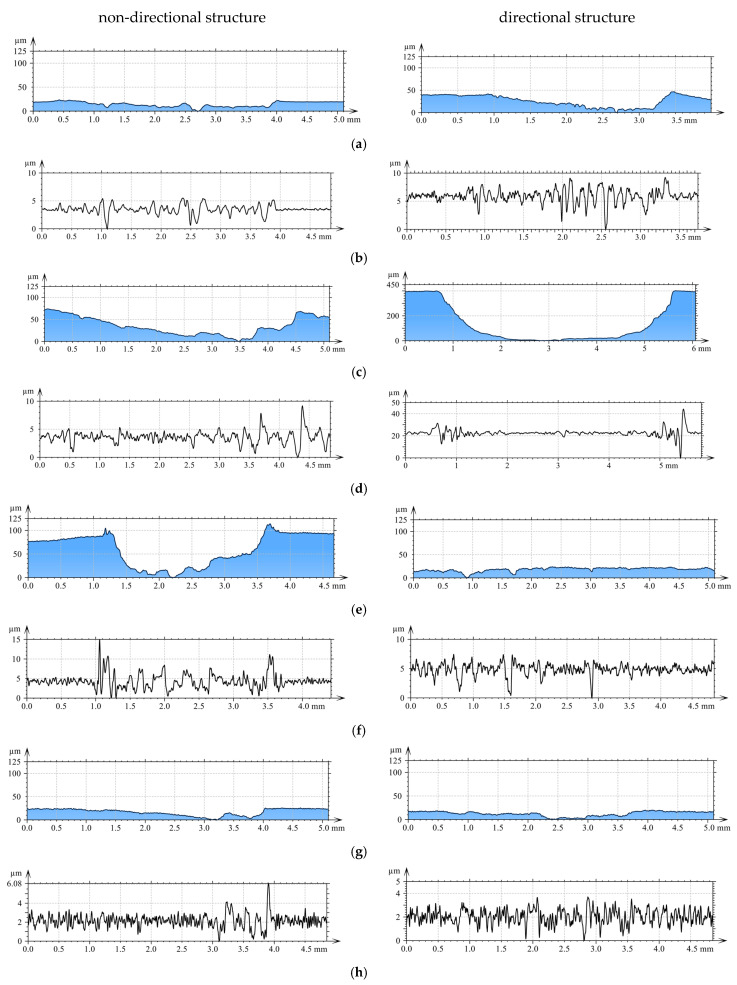
Surface roughness analysis of wear tracks: (**a**,**c**,**e**,**g**) roughness distribution in selected cross-sections perpendicular to friction direction for (**a**,**b**) Cu-ETP; (**c**,**d**) AlSi12CuNiMg alloy; (**e**,**f**) AlSi12CuNiMg + SiC composite; (**g**,**h**) 1.3505 steel; (**b**,**d**,**f**,**h**) roughness profiles (Gaussian filter, 0.25 mm cutoff).

Supplementary profilometric investigations facilitated both quantitative and qualitative evaluation of wear traces developed on the examined surfaces (Figure 5). Figure 5 shows the roughness profile curve and roughness (using a 0.25 mm Gaussian filter) at a representative location of the cross-section perpendicular to the friction direction.

In Figure 5, for the directional pin structure, the highest total abrasion depth was observed for AlSi12CuNiMg alloy at approximately 405 µm (Figure 5c). The smallest wear depth values were obtained for AlSi12CuNiMg + SiC composite (Figure 5e) and for 1.3505 steel (Figure 5g)—18.9 µm and 16 µm, respectively. This is confirmed by roughness distributions in the Gaussian filter, where for AlSi12CuNiMg + SiC composite—7.45 µm (Figure 5f), while for 1.3505 steel a value of 3.5 µm was obtained (Figure 5h).

Analysis of wear traces on matrix materials following interaction with cast iron pins revealed complex surface geometries. At the bottom of wear traces, discontinuous furrows were observed, interrupted by regions of significantly deformed material (Figure 5a,c,e,g). In Figure 5c, for directional pin structure, grooves at the bottom were uniformly distributed with evidence of transition to abrasive wear mechanisms.

In Figure 5e, for non-directional pin structure, the grinding surface appearance is characteristic of adhesive wear mechanisms. Large furrows created in the specimen were closed by deformed and displaced material during friction. In the upper portion of the wear trace, material was elevated above the primary surface. The total abrasion depth was 0.12 mm, including height above the primary surface (0.1 mm) and wear depth (0.02 mm).

### 3.2. Coefficient of Friction

**Figure 6:**
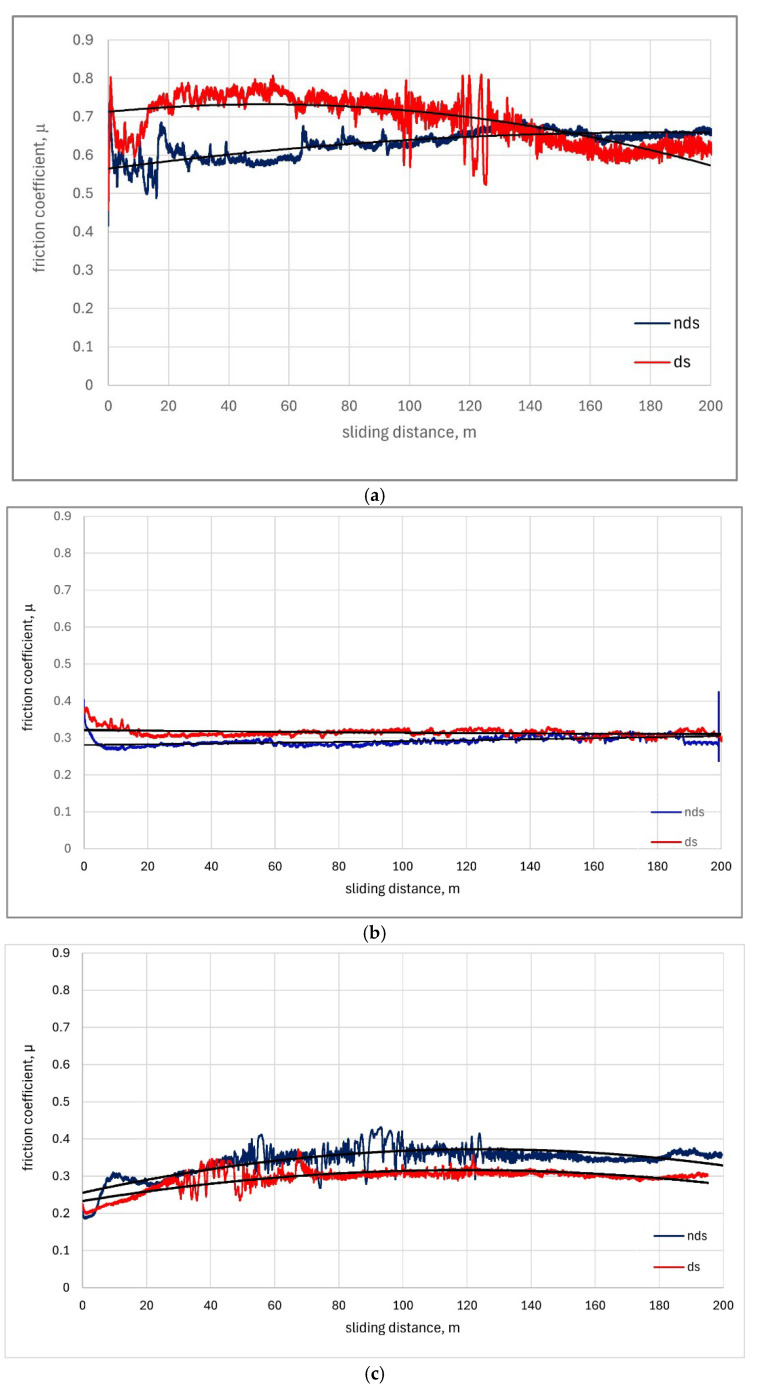

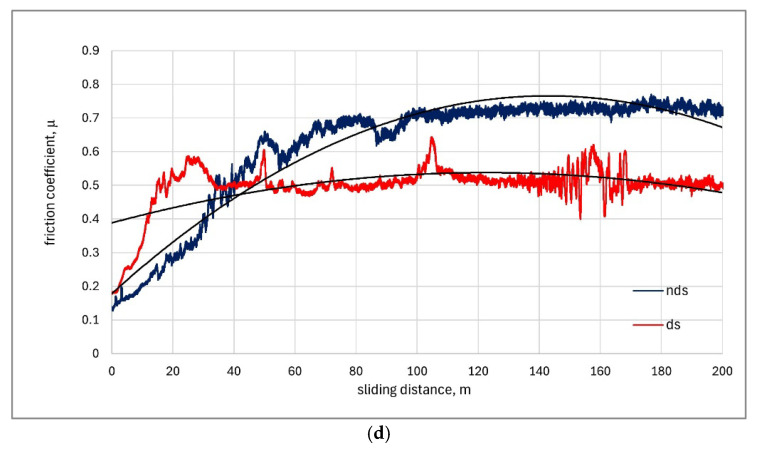
Friction coefficient (μ) versus sliding distance for tested materials under dry sliding conditions: (**a**) Cu-ETP; (**b**) AlSi12CuNiMg alloy; (**c**) AlSi12CuNiMg + SiC composite; (**d**) 1.3505 steel, black line—trends lines.

The coefficient of friction as a function of sliding distance for tested materials is shown in Figure 6. The compilation shows friction coefficient values recorded over distances from 0 to 200 m. The trend lines are marked with a solid black line.

For Cu-ETP (Figure 6a), friction coefficient changes were unstable for both directional and non-directional structure white cast iron, with average values exceeding 0.6 and 0.5, respectively. As observed, the friction coefficient value during the entire sliding distance for directional structure white cast iron changed from µ = 0.5 to µ = 0.7 and for non-directional structure from µ = 0.5 to µ = 0.8. Such high variation (equal to 0.2) is unacceptable. Up to sliding distance of approximately 150 m, friction coefficient values were lower for non-directional structure white cast iron, while above this distance values were higher than for directional structure white cast iron.

For AlSi12CuNiMg alloy as the block in the friction pair, friction coefficient changes were stable for both directional and non-directional structure white cast iron, with an average value of approximately µ = 0.3 (Figure 6b). For non-directional structure white cast iron, values were slightly lower throughout the sliding distance from µ = 0.27 to µ = 0.3. Trend curves for both structures are very close to horizontal lines.

For the AlSi12CuNiMg + SiC composite (reinforced with SiC carbide) used as the friction pair block material (Figure 6c), friction coefficient values throughout the sliding distance were lower for directional structure and ranged from µ = 0.2 to µ = 0.37. For directional structure, similar unstable friction coefficient changes were observed up to 75 m sliding distance and for non-directional structure up to 125 m sliding distance. From 125 to 200 m, the friction coefficient achieved stability.

For steel 1.3505 (Figure 6d), the friction coefficient was lower for directional structure cast iron. A rapid increase from µ = 0.2 to µ = 0.6 was observed over a distance of up to 25 m. Subsequently, stabilization occurred at approximately µ = 0.5 up to 200 m. For non-directional structure, a rapid increase from µ = 0.15 to µ = 0.7 was observed over a distance of up to 70 m and stabilization at µ = 0.7, with increases and decreases in the range of µ = 0.4 to µ = 0.65.

The instability regions visible in the graphs (Figure 6a,d) showing friction coefficient variations (0.2 range) observed during interaction with directionally solidified cast iron pins occur after sliding distances exceeding 100 m. Considering the statistical analysis methodology applied to the experimental data, these anomalies indicate periodic phenomena occurring between the sliding surfaces. The working surface of the pin likely undergoes changes that subsequently disappear (similar to a running-in process) after approximately 10 m of sliding distance (roughly 850 cycles). Complete explanation of these friction-related phenomena will be the subject of the authors’ future investigations. Similar instabilities appearing in the initial stages of the friction process can be attributed to the running-in of mating surfaces, and this process shows minimal dependence on the types of sliding materials involved.

### 3.3. Roughness Parameters

**Figure 7:**
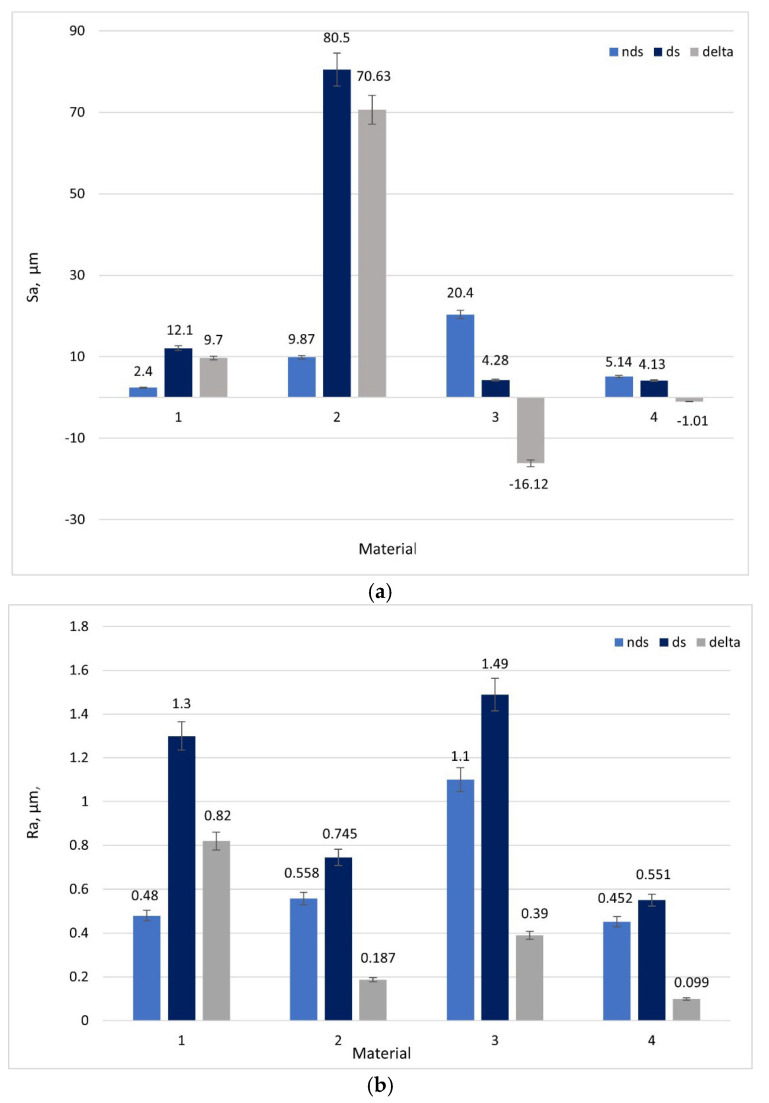
Surface roughness parameters: (**a**) Sa; (**b**) Ra values of wear tracks for tested materials: 1- Cu-ETP, 2—AlSi12CuNiMg alloy, 3—AlSi12CuNiMg + SiC composite, 4—1.3505 steel; nds—non-directional structure, ds—directional structure, delta—difference between ds-nds values.

Based on 3D images (Figure 4), it can be concluded that all tested materials exhibited abrasive wear mechanisms. However, significant differences in intensity were observed that can be compared using selected parameters. To evaluate surface geometry changes due to friction, basic roughness parameters were utilized, including (Sa)—arithmetical mean surface height (Figure 7a) and the arithmetical mean roughness value (Ra)—calculated as the arithmetical mean of absolute values of profile deviations from the mean line of the roughness profile (Figure 7b). Delta represents the parameter difference between directional and non-directional pin structures.

For blocks manufactured from Cu-ETP and AlSi12CuNiMg alloy, the Sa parameter (Figure 7a) showed higher values for pins made with directional white cast iron structure. Differences between values for directional and non-directional structures were: ΔSa = 9.7 µm and ΔSa = 70.3 µm, respectively. For blocks made from AlSi12CuNiMg+SiC composite and steel 1.3505, Sa parameters showed higher values when cooperating with non-directional white cast iron structure pins. Differences between values for directional and non-directional structures were: ΔSa = −16.12 µm and ΔSa = −1.1 µm, for AlSi12CuNiMg+SiC composite and 1.3505 steel, respectively. The highest Sa parameter value for directional structure pins was observed for AlSi12CuNiMg alloy at Sa = 80.5 µm.

For all four materials used as blocks in friction pairs, higher Ra parameter values were observed for directional structure white cast iron pins (Figure 7b). The highest Ra = 1.49 µm value was obtained for AlSi12CuNiMg + SiC composite, followed by Cu-ETP, for which the parameter difference for directional and non-directional structures was greatest (ΔRa = 0.82 µm, Figure 7b). The smallest differences in ΔRa = 0.099 µm parameter value for directional and non-directional structure white cast iron were observed for the 1.3505 steel block.

The absence of statistically significant differences in surface geometry parameters Ra and Sa after friction, observed in the results obtained from friction testing of cast iron against the steel block, demonstrates the high wear resistance of steel relative to the other investigated friction pairs. Over the tested distance of 200 m (regardless of the white cast iron structure employed: directional or non-directional), the wear level measured by wear track depth (Figure 5g) and surface geometry parameters Ra and Sa (Figure 7) is markedly lower than that of copper and aluminum alloys and shows no dependence on the white cast iron structure used. It is possible that employing a longer friction distance would reveal differences, but this represents a research task requiring a separate series of investigations. Paired t-test analysis revealed that differences in Sa parameters between directional and non-directional pins were statistically significant for Cu-ETP (*p* < 0.001), AlSi12Mg (*p* < 0.01) and AlSi12Mg + SiC (*p* < 0.001). For steel 1.3505, the difference ΔSa = −1.1 µm did not achieve statistical significance (*p* = 0.12), indicating comparable surface heights for both structures. Statistical analyses were performed using OriginPro 2023 software.

## 4. Results Discussion

### 4.1. Comparison with Tribological Studies of White Cast Iron

The friction coefficients recorded in this study for directionally solidified cast iron, ranging from μ = 0.30 to 0.70, are in agreement with previously published values for metallic-cast iron tribological systems. For instance, investigations by Krawczyk [7] into mottled cast iron with varied chemical compositions documented friction coefficients between 0.29 and 0.40 during dry sliding, which is a close match to our findings for pairs involving AlSi12CuNiMg alloy and 1.3505 steel. The observed reduction in friction for the directional structure when paired with harder materials, such as the AlSi12CuNiMg+SiC composite and 1.3505 steel, is attributed to the improved load-bearing capacity of the aligned cementite plates. This aligns with observations made by Fashu and Trabadelo [8] in their work on high-chromium white cast irons exposed to wear-corrosive conditions.

Furthermore, the measured wear depths, which spanned from 16 to 405 μm, are comparable to those documented in the recent literature concerning directionally solidified cast materials. Purba et al. [9] in their study of three-body abrasive wear in multicomponent white cast irons, reported similar wear depth ranges, thereby reinforcing the conclusion that microstructural orientation is a significant factor in wear resistance. The superior performance of the directional structure with certain material pairings in our work lends support to the conclusions of González-Pociño et al. [10] who showed that controlled microstructural alignment in white cast iron could diminish erosive wear by as much as 30% under ideal circumstances.

### 4.2. Dependence of Wear Mechanisms on Microstructure

The discrepancies in tribological behaviour between the directional and non-directional structures can be accounted for by the distinct wear mechanisms that operate within each material pairing. An analysis of the wear tracks, presented in Figure 5, reveals that abrasive wear is the dominant mechanism across all tested configurations. This observation is consistent with the findings of Barutçuoğlu et al. [11] for Ni-hard white cast irons alloyed with tungsten and boron.

In the case of the directional structure, the cementite plates within the ledeburite are aligned parallel to the direction of solidification, acting as reinforcing elements that enhance resistance to the penetration of abrasive particles. A similar reinforcement mechanism was reported by Zheng et al. [26] for cementite-iron composites, where they found that higher cementite volume fractions (>65%) markedly improved wear resistance by facilitating load transfer from the softer pearlite matrix to the hard cementite plates.

The microstructural advantage conferred by directional solidification is most evident when considering the composite nature of white cast iron. As demonstrated by Umemoto and Ohtsuka [25] the primary resistance to wear is provided by the hardness of the cementite (760–1340 HV), while the pearlite matrix (180–220 HB) contributes ductility. In directionally solidified structures, the continuous alignment of the cementite phase establishes preferential pathways for load distribution, which in turn reduces stress concentrations and minimizes the initiation and propagation of cracks.

### 4.3. Analysis of Performance Specific to Material Pairs

The superior performance of the directional structure when tested against the AlSi12CuNiMg+SiC composite and 1.3505 steel is a consequence of complementary hardness relationships and compatible wear mechanisms. With the AlSi12CuNiMg + SiC composite, the hard SiC particles (2500–3000 HV) induce a three-body abrasion scenario. In this context, the aligned cementite plates of the directional cast iron offer greater resistance to particle-induced gouging than randomly oriented structures. This outcome aligns with research by Jokari-Sheshdeh et al. [12] on Al-SiC composites, which demonstrated that wear resistance is enhanced when the alignment of the harder phase corresponds with the sliding direction. The 16.12 μm decrease in the Sa parameter for the directional structure (Figure 7a) signifies a more uniform wear distribution, suggesting that the oriented cementite plates are more effective at deflecting abrasive particles.

Conversely, the better performance of the non-directional structure with the softer Cu-ETP and AlSi12CuNiMg alloy (Cu: ~100 HB, AlSi12CuNiMg: ~80 HB) is attributable to the prevalence of adhesive wear mechanisms, as evidenced by the material transfer and surface deformation visible in Figure 5. The random orientation of cementite in the non-directional structure creates multiple points of resistance against the formation of adhesive junctions, a mechanism comparable to that reported by Sudhakar et al. [13] for high-chromium cast iron sliding against soft aluminum alloys. The contact pressure distribution between harder materials (1.3505 steel, AlSi12CuNiMg + SiC) and the cast iron establishes conditions where oriented carbides can effectively resist penetration. According to Jobayer Huq et al. [14], the critical element is the alignment of hard phases relative to the stress field, which explains why the directional structure excels with harder counterparts but performs poorly with softer materials where stress distributions differ.

### 4.4. Practical Implications and Limitations of the Study

The material-dependent performance of directional versus non-directional white cast iron structures carries significant implications for industrial applications. For bearing surfaces intended to operate against hard counterparts like steel or ceramic composites, directionally solidified white cast iron inserts could offer an extended service life due to lower friction and enhanced wear resistance. However, for applications involving softer metallic counterparts, conventional casting methods may prove more beneficial.

It is important to acknowledge several limitations of the present study. First, the tests were performed at an ambient temperature of 25 ± 1 °C, whereas numerous practical applications entail elevated temperatures where thermal effects can significantly alter microstructure and mechanical properties [15]. Second, the sliding distance of 200 m might not be sufficient to characterize long-term wear behaviour, especially for material systems known to exhibit transitional wear regimes. Finally, the specific directional solidification parameters used (withdrawal rate: 167 μm/s, temperature gradient: 33.5 K/mm) yield a particular microstructural scale and orientation that may not be optimal for all tribological contexts. Future work should therefore focus on investigating the effects of varying these solidification parameters on tribological performance under a wider array of operating conditions.

## 5. Conclusions

The tribological investigation conducted under dry friction conditions enabled the evaluation of material behaviour under extremely adverse operating conditions.

From the perspective of cast iron pin structure, directional structure orientation influences cooperation more favourably with blocks consisting of AlSi12CuNiMg + SiC composite and steel 1.3505. Smaller wear traces were observed compared to non-directional structure pins. Lower friction coefficient values were achieved: average values µ = 0.30 and µ = 0.40. Lower Sa parameter values were observed: Sa = 4.28 and Sa = 4.13. Roughness distribution in selected cross-sections was also characterized by smaller values: 18.9 µm and 16 µm, respectively. The smallest values were also obtained for wear profile curves for AlSi12CuNiMg + SiC composite and steel 1.3505 (18.9 µm and 16 µm, respectively).

For friction pairs: white cast iron pin—Cu-ETP block and AlSi12CuNiMg alloy—non-directional structure was more favourable from the pin structure perspective. This is confirmed by comparing obtained images of surface geometry after friction. The roughness parameters were also lower for Cu-ETP and AlSi12CuNiMg alloy (Sa = 2.4 and Sa = 9.87, respectively) than for directional structure pins. Roughness distribution was also lower for non-directional structure pins and amounted to 17.0 µm for Cu-ETP and 57.6 µm for AlSi12CuNiMg alloy.

Based on conducted investigations, it cannot be unambiguously stated that pins with directional white cast iron structure perform better in friction pairs than pins with non-directional structure. Research results indicate that the cooperating material in the friction pair is significant. In the conducted studies, two out of four applied block materials, namely AlSi12CuNiMg + SiC composite and steel 1.3505, produced more favourable effects for directional structure cast iron pins: lower friction coefficient µ and greater wear resistance.

Taking the above findings into account, further validation studies should be undertaken, including the use of additional block materials, in order to expand the understanding of the potential applications of directionally solidified pins made of eutectic white cast iron.

## Figures and Tables

**Table 1:** Chemical composition, wt. %.

**C**	**Si**	**Mn**	**P**	**S**	**Cr**	**Ni**	**Mo**	**Al**	**Cu**
4.25	0.058	0.64	0.0079	0.021	0.033	0.0093	<0.0020	0.011	0.032
**Co**	**Ti**	**Nb**	**V**	**W**	**Pb**	**Mg**	**B**	**Sn**	**Zn**
0.0024	<0.001	<0.0040	0.0022	<0.010	<0.0030	<0.0010	0.0009	0.0061	<0.002
**As**	**Bi**	**Ca**	**Ce**	**Zr**	**La**	**Fe**			
0.0069	<0.002	0.0005	<0.0030	0.0043	0.0013	94.9			

**Table 2:** Chemical composition, wt. %.

	C	Si	Cu	Mg	Ni	Mn	Ti	O	SiC	Al	Cr	Fe
Cu-ETP		-	≥99.9	-	-	-	-	≤0.04	-	-	-	-
AlSi12CuNiMg		12	1.0	1.0	0.9	0.2	0.05	-	-	bal.	-	-
AlSi12CuNiMg + SiC		11.5	2.0	2.0	0.9	0.2	0.05	-	15.0	bal.	-	-
1.3505	0.95	0.15	-	-	0.3	0.35	-	-	-	-	1.65	bal.

## Data Availability

The original contributions presented in this study are included in the article. Further inquiries can be directed to the corresponding author.
